# Automated sample-changing robot for solution scattering experiments at the EMBL Hamburg SAXS station X33

**DOI:** 10.1107/S0021889808021018

**Published:** 2008-08-16

**Authors:** A. R. Round, D. Franke, S. Moritz, R. Huchler, M. Fritsche, D. Malthan, R. Klaering, D. I. Svergun, M. Roessle

**Affiliations:** a EMBL Hamburg, Building 25a, Notkestrasse 85, 22603 Hamburg, Germany; b Fraunhofer Institute for Manufacturing, Engineering and Automation IPA, Department of Production and Process Automation, Nobelstrasse 12, 70659 Stuttgart, Germany; c Institute of Crystallography, Russian Academy of Sciences, Leninsky prospekt 59, 117333 Moscow, Russian Federation

**Keywords:** automation, high throughput, small-angle X-ray scattering (SAXS), solution, biological macromolecules

## Abstract

There is a rapidly increasing interest in the use of synchrotron small-angle X-ray scattering (SAXS) for large-scale studies of biological macromolecules in solution, and this requires an adequate means of automating the experiment. A prototype has been developed of an automated sample changer for solution SAXS, where the solutions are kept in thermostatically controlled well plates allowing for operation with up to 192 samples. The measuring protocol involves controlled loading of protein solutions and matching buffers, followed by cleaning and drying of the cell between measurements. The system was installed and tested at the X33 beamline of the EMBL, at the storage ring DORIS-III (DESY, Hamburg), where it was used by over 50 external groups during 2007. At X33, a throughput of approximately 12 samples per hour, with a failure rate of sample loading of less than 0.5%, was observed. The feedback from users indicates that the ease of use and reliability of the user operation at the beamline were greatly improved compared with the manual filling mode. The changer is controlled by a client–server-based network protocol, locally and remotely. During the testing phase, the changer was operated in an attended mode to assess its reliability and convenience. Full integration with the beamline control software, allowing for automated data collection of all samples loaded into the machine with remote control from the user, is presently being implemented. The approach reported is not limited to synchrotron-based SAXS but can also be used on laboratory and neutron sources.

## Abstract 2

An automated sample changer for small-angle X-ray scattering (SAXS) on protein in solution is reported. The technical implementation and integration to a synchrotron-based SAXS beamline is described.

## 1. Introduction   

Data collection for any experimental technique can be tedious, laborious work, where repetitive measurements are required to be performed in a consistent way many times. For data collection using synchrotron radiation, there is the additional constraint of having to complete data collection within a limited timeframe dependent on the length of time allocated to the experiment. In order to reduce human errors, which are inherent in the taking of repetitive measurements, especially after working long hours, automation is required; this is especially important when dealing with biological samples, as they are often available in limited quantities and obtained after extensive preparative work. The necessity of automation has become even more apparent with the advent of third generation synchrotron sources, which have significantly reduced data collection times. Automatic sample changers have revolutionized the use of synchrotron sources, in particular in protein crystallography (Cohen *et al.*, 2002 ▶; Arzt *et al.* 2005 ▶) where high-throughout studies and remote (‘FedEx’) operation have become possible (McPhillips *et al.*, 2002 ▶).

The recent renaissance of the use of small-angle X-ray scattering (SAXS) for structure analysis in biology and biochemistry has prompted a rapidly increasing interest in large-scale studies of biological macromolecules in solution (Svergun & Koch, 2003 ▶; Petoukhov & Svergun, 2007 ▶). Solution SAXS experiments on a synchrotron can be performed within fractions of a second (undulator) or within a few minutes (bending magnet). Novel data analysis methods enable the comprehensive analysis of data and the construction of low-resolution structural models of individual macromolecules and functional complexes *ab initio* and in terms of available high-resolution fragments (Konarev *et al.*, 2006 ▶; Petoukhov & Svergun, 2007 ▶). Given that SAXS not only provides the static structure but also yields information about the structural transitions (*e.g.* due to ligand binding) and variations in physical and chemical parameters, such as pH, temperature, ionic strength *etc*., as well as allowing one to monitor kinetic processes (folding or assembly), automation of a SAXS experiment would be of immense value for the analysis of biomacromolecular solutions.

Perhaps the major difficulty in the automation of a solution SAXS experiment lies in the fact that obtaining the scattering pattern from the dissolved particles includes at least two independent measurements, from the solute and from the solvent, and the latter pattern has to be subtracted from the former one. This operation is required not only to remove the solvent scattering but also to get rid of the instrumental background. Biological objects containing light atoms are weak X-ray scatterers and the difference scattering between the solute and the solvent is often extremely low (the useful signal is only a fraction of a percent higher than the background). The measurements must therefore be performed in the same sample compartment to avoid even slight differences in the sample cell material (thickness, absorption *etc*). The high requirements of the sample cell prevent the use of automatic pre-filled and single-use sample containers such as standard well plates. As a consequence, the compartment must be thoroughly cleaned and dried before the loading of the next fluid sample. Cell cleaning, drying and loading are carried out manually on most SAXS stations, and this procedure may take a few minutes or more. Any high-throughput initiative in SAXS would therefore require an automated sample changer. Here we present a dedicated liquid-handling system for biological solution SAXS developed in collaboration between the EMBL (Hamburg Outstation) and the Fraunhofer Institute for Manufacturing, Engineering and Automation (IPA, Stuttgart) and report the first results of its user operation at the X33 beamline (storage ring DORIS-III, Hamburg).

## 2. Automatic sample-changer description   

**Figure 1:**
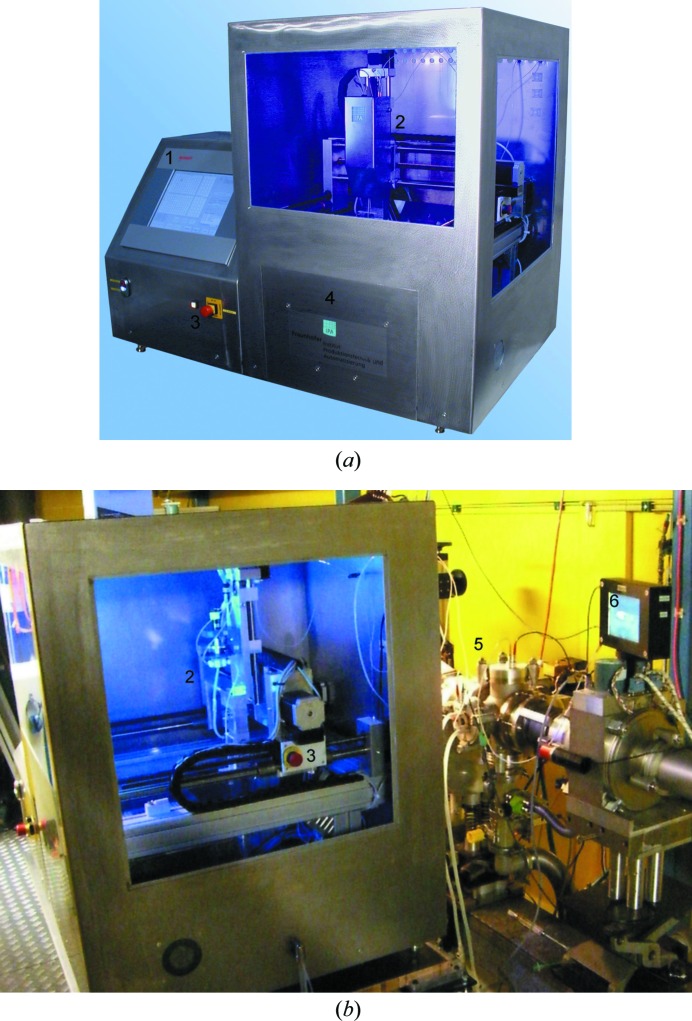
(*a*) Automatic sample changer. (*b*) In operation at the X33 beamline. (1) Integrated touchscreen monitor, (2) motorized *XYZ* stage, (3) emergency stop buttons, (4) sample storage drawer, (5) in-vacuum sample measurement environment, (6) video monitor to observe sample measurement cell.

The prototype device (Fig. 1 ▶) has been designed to fulfill all the tasks required to collect data according to the protocol in use at the X33 beamline. This bending-magnet beamline is dedicated to the measurement of solutions of biological macromolecules and has an in-vacuum sample compartment with flat mica or polycarbonate windows. The sample volume is about 40–45 µl and the typical exposure times are 1–2 min (Roessle *et al.*, 2007 ▶). The measuring protocol involves the loading of protein solutions and matched buffers in an identical way so as not to introduce errors; this approach requires the cleaning and drying of the sample cell between measurements.

**Figure 2:**
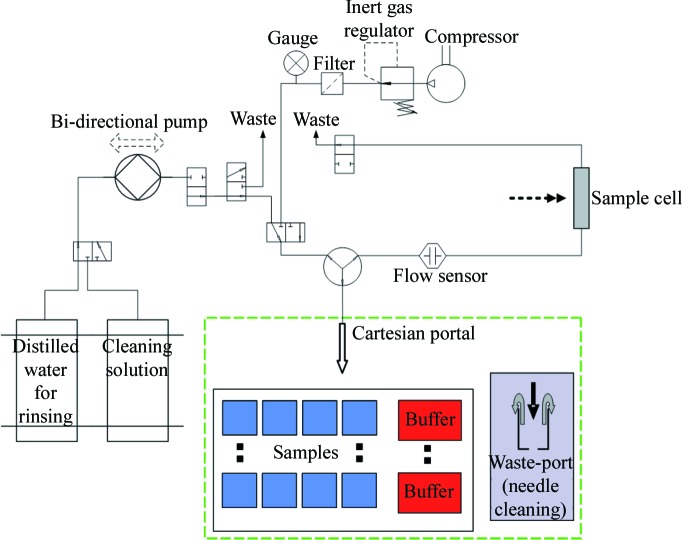
Schematic diagram of the automatic sample changer

Macromolecular solutions and matching buffers are held in a Peltier cooled storage tray which allows up to 192 samples (2 × 96 well plates for 200 µl Eppendorf tubes) to be held at temperatures in the range 273–333 K. Routine measurements currently underway using the prototype require 80–100 µl of solution, which is stored in sealed Eppendorf tubes with pierceable lids to prevent evaporation. The solution selected for measurement is extracted from the Eppendorf tube *via* a needle positioned using a motor-controlled *XYZ* translation stage and then transported to the (in-vacuum) measurement cell through standard 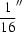 (1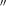 = 2.54 cm) polytetrafluoro­ethylene tubing (0.8 mm ID) used for high performance liquid chromatography systems (see Fig. 2 ▶). Sample verification is achieved using an optical flow sensor, which measures the attenuation of red light (λ = 650 nm) through the Teflon tubing. This measurement allows calculation of sample volume and direct determination of the sample position. The tube length from the sensor to the measurement cell is a known parameter, which ensures that the sample can be reliably positioned in the measurement cell for data collection. To ensure reliability for filling high-concentration protein samples, the internal diameter of all tubing and connectors is kept constant, reducing turbulence. However, the connections and valves may still cause disruptions to the flow and so transport speed is reduced while the sample is passing though valves to prevent foaming. The machine and measurement cell including all tubing and the needle are cleaned using a detergent and ethanol solution, rinsed with distilled water, before being dried with nitrogen. The time taken for loading the samples and the cleaning of the system is currently timed at 2 min and 2 min 30 s, respectively. However, it has been observed that drying accounts for the largest proportion of this cleaning time, and this is dependent on the temperature of the gas used to dry the system as increased temperature promotes evaporation of the residual water in the tubes and measurement cell. To ensure there is no cross contamination, the machine is flushed with 2 ml of cleaning solution and water, both passed through the system at a flow rate of 6 ml s^−1^.

**Figure 3:**
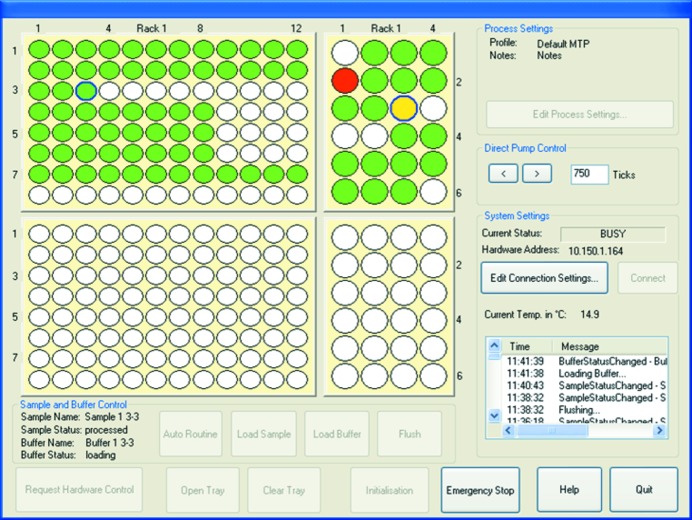
Screenshot of the graphical user interface during user operation

The prototype has been mounted on a guide rail with wheels and can be easily disconnected from the vacuum cell and moved out of the operational position by a single person. This allows for a quick changeover to a manual filling mode for special liquids, *e.g.* those with high viscosity. The prototype is equipped with an integrated (touchscreen) computer (Fig. 1 ▶ *a*), which allows for direct local control through the graphical user interface (GUI) (Fig. 3 ▶).

## 3. Control software   

A client–server-based network protocol has been employed so that the sample changer may be controlled either directly on the embedded computer, as described above, or from any computer within the same network. At any given time, only one user may have full control over the hardware; any other client may display the current status only.

The server directly operates the prototype, receiving and interpreting high-level user commands. These commands are translated to corresponding motor and valve control sequences. Furthermore, the server stores the machine, sample and buffer states, which can be retrieved *via* the network interface.

Currently, the client is implemented as a GUI, which is shown in Fig. 3 ▶. The GUI displays the well-plate positions of all samples and buffers (small and large circles, respectively). The colour of each well indicates its status: white, not processed; blue, measuring (not shown in Fig. 3 ▶); green, successfully loaded; yellow, processing; and red, indicating that the loading procedure failed. The failure to load correctly (at rack 1 position 1–2 in Fig. 3 ▶) was an expected indication that there was no solution to measure as there was no buffer placed in that position. The action to load buffer or sample is carried out on the currently highlighted position (circled in blue). The status of the highlighted sample and buffer is shown in the bottom left corner while the status of the machine can be seen on the right.

The client–server design also allows for scriptable and programmable modes of operation, implemented using the control system TINE (threefold integrated networking environment), developed at DESY (Bartkiewicz & Duval, 2007 ▶). The interface provided by TINE has, up to now, been employed to run test sequences automatically, but it can also be used to embed hardware control into high-level scripting languages such as Python. We are currently working on combining the control of the automatic sample changer with the data acquisition software controlling the detector at X33, to give an integrated interface for the users and to automate the measurement sequence fully.

## 4. Performance   

**Figure 4:**
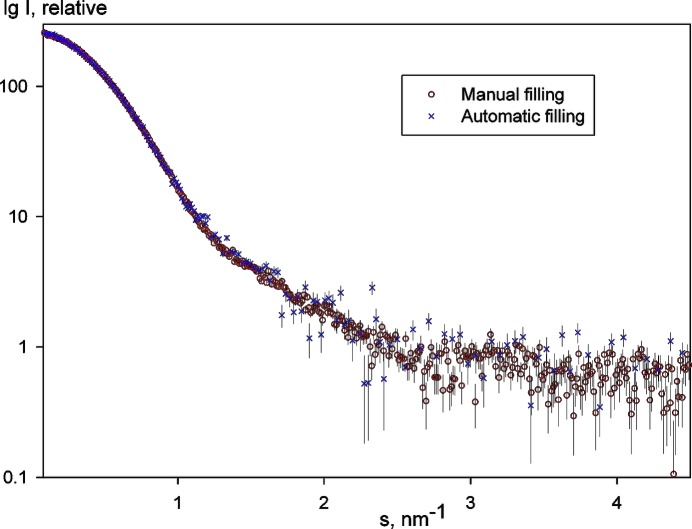
Comparison of 5 mg ml^−1^ BSA (bovine serum albumin) protein solution manually and automatically filled by the sample changer. The deviations are in the range of the experimental errors

The automatic sample changer was put into test operation in Hamburg in August 2007 and was thoroughly tested offline before installing it at the X33 beamline. Comparison of SAXS patterns between manual filling and automated operation showed no differences (see Fig. 4 ▶). During the first three months of user operation in September–December 2007, over 50 international groups used the changer to measure over 2000 protein samples of various concentrations. A total of over 4500 individual SAXS images have been collected at the X33 beamline from the samples loaded by the device.

Feedback from users indicates that current operational efficiency has been greatly increased using the automated sample changer compared with manual loading. The automated loading is more reliable and the time not spent changing the samples can be used for further sample preparation or data analysis. Initial reliability tests of filling using water showed a failure rate of less then 0.7% (2 in 300 repetitions). Following optimization, the tested failure rate using the automatic loading script developed as part of the integrated data acquisition program was less than 0.5%. The small number of failures is due to bubbles forming in the measurement cell, preventing correct measurement of the solution.

**Figure 5:**
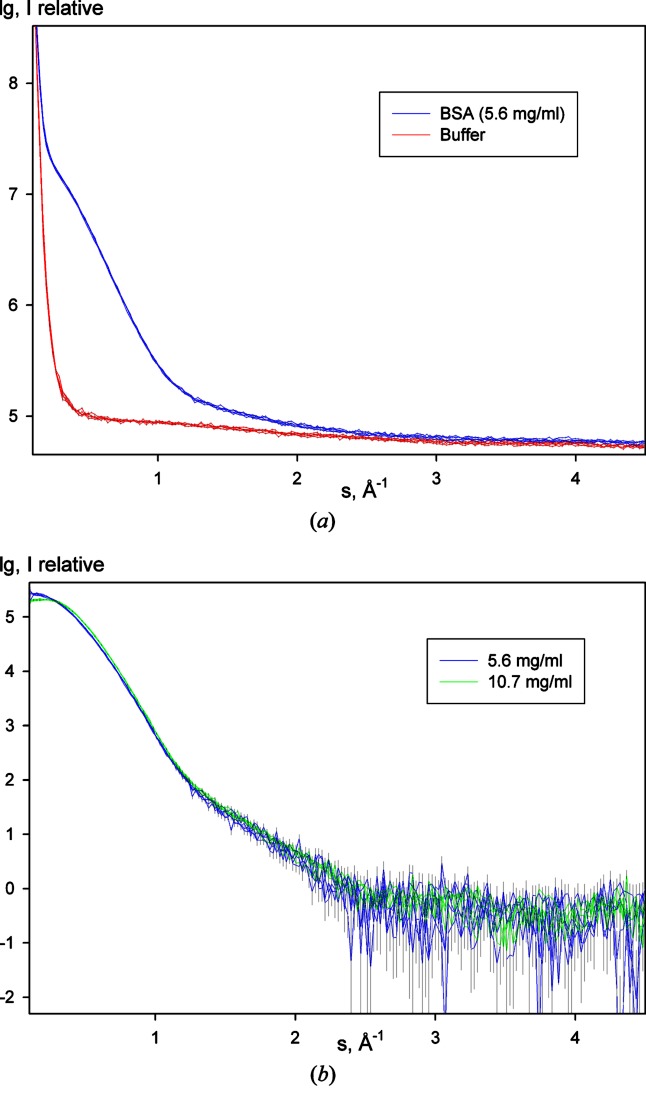
(*a*) Raw data collected from measurements of five samples of 5.6 mg ml^−1^ BSA (blue) and five buffers (red) showing no contamination of protein in buffer measurements. (*b*) Subtracted scattering data from 5.6 mg ml^−1^ (blue) and 10.7 mg ml^−1^ (green) BSA; multiple measurements of samples of like concentrations match within calculated errors (from Poisson counting statistics) and the difference between the two concentrations is attributed to the inter-particle effects altering with concentration.

The tests confirmed that even rather viscose solutions (up to 150 mg ml^−1^ of lysozyme) were successfully loaded, and also samples dissolved in ethanol and methanol could be handled by the changer. The automatic cleaning procedure implemented by the prototype has shown there is no cross contamination of samples (see Fig. 5 ▶) as stability of measurements has improved. In addition, the useable lifetime of the measurement cell has been increased by improved cleaning. With manual filling the windows of the measurement cell were replaced after two days’ use (approximately 150 measurements), while when using the automatic sample changer the measurement cell windows only need to be replaced every seven days (approximately 500 measurements).

## 5. Conclusions   

We report here the use of the sample changer in an attended mode, where the users monitor the measurement process (either on-site or remotely). Currently, the presence of bubbles in the measurement cell is classed as failure to load automatically as user intervention is required to correct the problem. Loading failures can be corrected by the users using direct activation of the pumps *via* the GUI and monitoring of the filling *via* a video monitor observing the measurement cell.

At present, the changer operates with 80–100 µl sample volume, which is about twice the cell volume. This safety margin is required to ensure bubble-free loading given the losses of the protein on the tube walls, and to allow the sample to flow through the cell during measurement to irradiate the fresh sample and thus reduce the radiation damage. The sample flow can also be initiated *via* the direct pump control.

Systems for automated loading of solutions for X-ray scattering measurements are being developed, for example, at the SYBYLS beamline (Berkeley, USA), the 4–2 station at SSRL (Stanford, USA), the SWING beamline at Soleil (Paris, France) and the BioCAT beamline at APS (Argonne, USA). The sample changer presented here is, to our knowledge, the first system in a heavy-duty day-and-night operation. Over 50 external user groups during a period of several months have used the sample changer, and this has allowed us to assess the efficiency gain due to automation. We deliberately started in an attended operation mode to obtain more experience regarding the reliability and convenience of the use of the device. Already the attended mode has become a major step forward in the automation of the experiment, improving significantly the ease of use and reliability of the user operation at the beamline. We plan to implement unattended operation by including a secondary monitoring system using the output of the video camera at the measurement cell and image recognition tools to ensure correct sample loading. Further optimization of the changer (in particular, by shortening the tubing) and inclusion of a heating system for the drying gas are intended, in order to decrease the time taken for loading the samples and cleaning of the system. Automatic sample cell filling could also be performed at X-ray laboratory sources and neutron facilities. The sample throughput on such experimental setups is limited by exposure time, and reliable day-and-night operation will improve the performance.
